# Letter to Editor: “Serum Vitamin D Levels and Its Relationship With Keloid, Acne or Hypertrophic Scar: A Two‐Sample Mendelian Randomization Study”

**DOI:** 10.1111/jocd.71176

**Published:** 2026-09-02

**Authors:** Shanzhong Qu, Yao Ni, Wenlong Tang, Xuhong Yan, Jie Zhang, Xiaoding Cheng

**Affiliations:** ^1^ Department of Dermatovenereology, West China School of Medicine Sichuan University, Sichuan University Affiliated Chengdu Second People's Hospital, Chengdu Second People's Hospital Chengdu, Sichuan China

To the Editor,

1

We read with interest the two‐sample Mendelian randomization (MR) study by Ji et al. [1], which investigated the potential causal effects of genetically predicted serum 25‐hydroxyvitamin D levels on keloids, acne, and hypertrophic scars. Although no significant associations were identified, these null findings should be interpreted cautiously because of the use of earlier outcome genome‐wide association study (GWAS) datasets, the lack of external validation, and the restriction to European‐ancestry populations.

First, the selection of the outcome GWAS datasets may have limited the statistical power of the analyses. The reliability of MR depends not only on the analytical methods but also on the suitability, sample size, and currency of the GWAS datasets. In particular, the numbers of cases, controls, and available variants directly affect the precision of SNP–outcome estimates and the power of causal inference [2]. The use of datasets with limited case numbers may therefore increase the risk of false‐negative findings. Ji et al. used earlier FinnGen releases for acne and hypertrophic scars, whereas FinnGen has since been updated to release 12 (R12). Compared with the datasets used in the original study, the R12 datasets contain substantially more cases, controls, and analyzable SNPs. For acne and hypertrophic scars, the numbers of cases in R12 are approximately 2.70–3.55 times higher, while the numbers of controls are approximately 2.24–2.26 times higher (Table 1). These increases may improve the precision of SNP–outcome associations and the statistical power of MR analyses. Therefore, the reported absence of associations between vitamin D and acne or hypertrophic scars may partly reflect insufficient power rather than definitive evidence of no causal effect. Reanalysis using the latest FinnGen release would provide a more reliable assessment.

**TABLE 1:** Comparison of GWAS sample sizes between the FinnGen release used in Ji et al. and FinnGen R12.

Trait	FinnGen release used in Ji et al.			FinnGen R12			Fold increase in cases
	cases	controls	SNPs	cases	controls	SNPs	
Hypertrophic scar	766	207 482	16 380 443	2068	465 673	20 112 393	2.7
Acne	1299	211 139	16 380 454	4617	476 404	20 112 531	3.55

2

Second, the study relied on a single outcome data source without independent replication. GWAS estimates may vary across databases because of differences in sample composition, case definitions, recruitment procedures, genotyping platforms, and phenotype ascertainment. External validation is particularly important when the number of cases is limited and the primary results are null, as it helps distinguish a true absence of effect from inadequate statistical power. If suitable summary statistics are available, the analyses could be replicated using resources such as the Million Veteran Program [3] or other large biobanks. Consistent findings across independent datasets, followed by meta‐analysis of the MR estimates, would strengthen the credibility of the conclusions.

Third, the exclusive use of European‐ancestry data limits the generalizability of the findings. Keloids and hypertrophic scars show substantial population variation and are more common in individuals of African and Asian ancestry. Vitamin D status and metabolism are also influenced by skin pigmentation, ultraviolet exposure, genetic background, and vitamin D receptor‐related pathways. Consequently, null findings in European populations may not apply to higher‐risk non‐European populations. Non‐European GWAS resources are increasingly available. GWAS data for vitamin D levels have been reported in East Asian, African, and South Asian populations, including GCST90474455, GCST90692019, and GCST90692128. Acne GWAS datasets are also available for South Asian and African populations, including GCST90727047 and GCST90478841. Although some of these datasets remain relatively small, they may still provide useful evidence for cross‐ancestry validation. Trans‐ethnic Mendelian randomization (TEMR) may therefore be an appropriate complementary approach [4]. By incorporating ancestry‐specific allele frequencies, linkage disequilibrium structures, and genetic effect estimates, TEMR can assess whether causal effects are consistent across populations. For phenotypes with marked population differences, such as vitamin D levels, keloids, acne, and hypertrophic scars, cross‐ancestry analyses could improve the generalizability of the findings and determine whether the null associations observed in European populations extend to Asian, African, and South Asian populations.

In conclusion, we appreciate the contribution of Ji et al. to the investigation of vitamin D and inflammatory or scar‐related skin disorders. However, before analyses using updated outcome datasets, independent replication, and cross‐ancestry validation are undertaken, the findings should be interpreted as an absence of significant genetic evidence in the currently analyzed European‐ancestry datasets rather than as definitive evidence against causal relationships.

## Funding

The authors have nothing to report.

## Ethics Statement

The authors have nothing to report.

## Consent

The authors have nothing to report.

## Conflicts of Interest

The authors declare no conflicts of interest.

## Data Availability

Data sharing not applicable to this article as no datasets were generated or analysed during the current study.
